# Bibliometric insights from publications on subchondral bone research in osteoarthritis

**DOI:** 10.3389/fphys.2022.1095868

**Published:** 2022-12-22

**Authors:** Pengfei Wen, Rui Liu, Jun Wang, Yakang Wang, Wei Song, Yumin Zhang

**Affiliations:** Department of Joint Surgery, Honghui Hospital, Xi’an Jiaotong University, Xi’an, Shaanxi, China

**Keywords:** subchondral bone, osteoarthritis, knowledge landscape, bibliometric analysis, hotspots, research trends

## Abstract

**Background:** The role of subchondral bone in the pathogenesis of osteoarthritis has received continuous attention worldwide. To date, no comprehensive bibliometric analysis of this topic has been carried out. The purpose of this study was to investigate the knowledge landscape, hot spots, and research trends in subchondral bone research through bibliometrics.

**Methods:** Web of Science Core Collection database was used to collect articles and reviews on subchondral bone in osteoarthritis published between 2003 and 2022. CiteSpace, VOSviewer, Scimago Graphica, and a bibliometric online analysis platform (http://bibliometric.com/) were used to visualize the knowledge network of countries, institutions, authors, references, and keywords in this field. Both curve fitting and statistical plotting were performed using OriginPro, while correlation analysis was done using SPSS.

**Results:** A total of 3,545 articles and reviews were included. The number of publications on subchondral bone showed an exponential growth trend. The US produced the most (980), followed by China (862) and the United Kingdom (364). Scientific output and gross domestic product were significantly correlated (*r* = .948, *p* < .001). The University of California System and Professor Pelletier Jean-Pierre were the most prolific institutions and influential authors, respectively. The most active and influential journal for subchondral bone research was Osteoarthritis and Cartilage. The majority of papers were financed by NSFC (474, 13.4%), followed by HHS (445, 12.6%), and NIH (438, 12.4%). In recent years, hot keywords have focused on the research of pathomechanisms (e.g., inflammation, apoptosis, pathogenesis, cartilage degeneration/repair, angiogenesis, TGF beta) and therapeutics (e.g., regeneration, stromal cell, mesenchymal stem cell).

**Conclusion:** Subchondral bone research in osteoarthritis is flourishing. Current topics and next research trends would be centered on the pathomechanisms of cellular and molecular interactions in the subchondral bone microenvironment in the development of osteoarthritis and the exploration of targeted treatment medicines for the altered subchondral bone microenvironment.

## 1 Introduction

Osteoarthritis (OA) is a common degenerative disease that affects more than 250 million people and is a major cause of disability, severely reducing the quality of life of patients and causing a significant socioeconomic burden (Hunter and Bierma-Zeinstra, 2019). Articular cartilage damage, abnormal subchondral bone remodeling, synovial inflammation, and ligament and meniscus degeneration are some of its pathological characteristics, which consequently lead to pain, joint dysfunction, and loss of tissue integrity (Dieppe and Lohmander, 2005; Hügle and Geurts, 2017). Current strategies for the management of OA are broadly divided into non-pharmacological, pharmacological, and surgical treatments (Kroon et al., 2018; Bannuru et al., 2019; Kloppenburg and Berenbaum, 2020). However, there are few satisfactory options for improving intra-articular homeostasis and decelerating OA progression. The subchondral bone, a layer of bone plate and trabecular bone beneath the articular cartilage, is a microscopic structural unit composed of a variety of cells, including bone cells, bone matrix, endothelial cells, immune cells, cytokines, and sensory neurons, which could provide mechanical and nutritional support to the cartilage. Some evidence suggested that alterations in the cellular and extracellular components of the subchondral bone microenvironment can cause abnormalities of subchondral bone remodeling, angiogenesis, and sensory innervation, thereby leading directly or indirectly to cartilage destruction and pain (Hu W. et al., 2021; Hu Y. et al., 2021). In addition, subchondral bone can be involved in the pathophysiology of OA by affecting cartilage degeneration through mechanical stress alterations or paracrine-mediated bone-chondral crosstalk (Zhen et al., 2013; Lin et al., 2019; Ni et al., 2020). Overall, the role of subchondral bone in the pathogenesis of OA has attracted extensive attention from researchers, and some promising therapeutic agents have been developed and tested *in vitro* and *in vivo*.

It is getting harder for researchers, particularly for fresh investigators, to completely comprehend, assess, and identify the most pertinent and significant information in the area as a result of the subchondral bone research literature’s rapid growth. Therefore, it is essential to give a macroscopic description of research trends, hot spots, and high-impact articles, institutions, and authors in this field. An increasingly prevalent way to get the aforementioned parameters is through bibliometric analysis. It has been heavily utilized in orthopedics (Zhang Y. et al., 2021; Yang et al., 2021) and other medical specialties (Wen et al., 2022a; Song et al., 2022) to promote the development of medical research and clinical practice. Hu et al. revealed 10 hot microRNAs of chondrocytes in OA and their potential targets based on bibliometrics (Hu et al., 2022). Yang et al (2022) performed a bibliometric analysis of the knowledge structure and trends in OA-related macrophage research. Zhang et al (2022) visualized the historical evolution and future trends of hyaluronic acid for OA. However, to our knowledge, no bibliometric studies on subchondral bone in OA have been reported. Therefore, this study conducted a comprehensive analysis of scientific publications on subchondral bone research over the past 20 years in order to determine the current research status and knowledge landscape and to help researchers understand historical hot spots and research trends in subchondral bone research.

## 2 Methodology

### 2.1 Data collection

**FIGURE 1:**
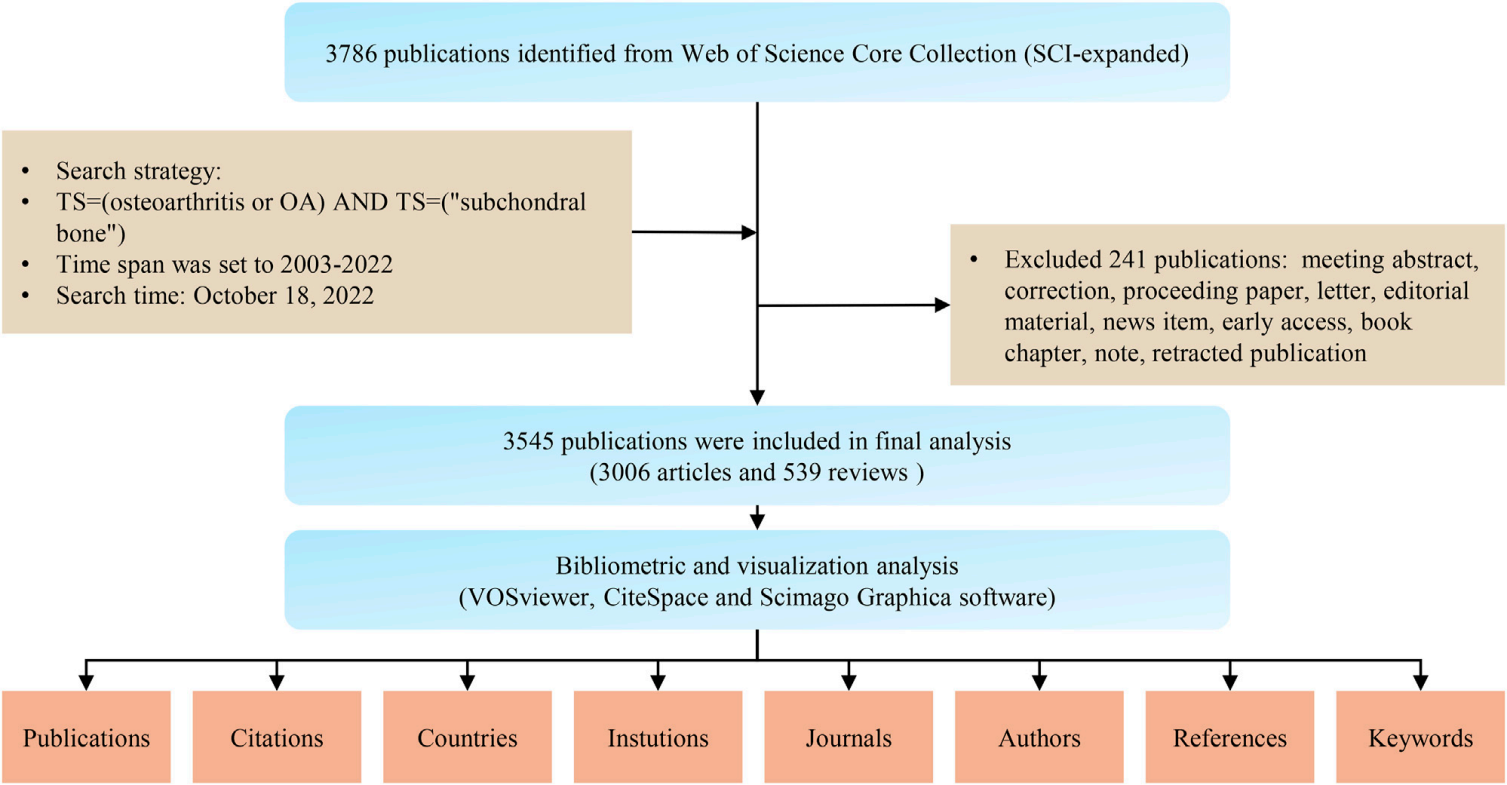
Flowchart of the literature search, screening, and analysis.

Web of Science (WoS) contains more than 12,000 international academic journals and is widely used for bibliometric study (Wu et al., 2021; Wen et al., 2022b). To avoid bias caused by database updates, all of the papers for our study was retrieved and downloaded on 18 October 2022, from the Science Citation Index Expanded of WoS Core Collection database. The specific search strategy was as follows: TS = (osteoarthritis or OA) and TS = (“subchondral bone”), with the time span set to 2003–2022. A total of 3,876 publications were retrieved, of which 241 invalid records were excluded. Finally, 3,545 valid publications were obtained in the final dataset for further analysis (Figure 1). The extracted data included authors, titles, publication years, citation times, countries, institutions, journals, highly cited articles, references, and keywords. Journal information including impact factor (IF) and quartile in categories (Q1, Q2, Q3, and Q4) was collected from the 2021 Journal Citation Report. The Hirsch index (HI) was obtained by WoS. Gross domestic product (GDP) data for each country were obtained from the official website of the World Bank (https://data.worldbank.org.cn/).

### 2.2 Data analysis

Visualization software, including VOSviewer (v.1.6.18), CiteSpace (5.8.R3), and Scimago Graphica, was used in this study. Scimago Graphica was used to map the global distribution of national publications. The online bibliometric analysis platform (https://bibliometric.com) was used to visualize the collaboration network of countries. VOSviewer was used to visualize the co-authorship analyses of institutions and authors, as well as the co-occurrence analysis of keywords. Finally, CiteSpace was used for reference co-citation analysis, keyword burst detection, and drawing timeline views of reference clusters. Statistical analysis was performed using IBM SPSS 22.0 (IBM Corp., Armonk, NY, United States). Microsoft Excel 2016 (Microsoft Corp., Redmond, WA, United States) was used to collate the data. Statistical plots and curve fitting were conducted using OriginPro 9.1 (OriginLab Corp., Northampton, MA, United States). The strength of the correlation between continuous variables was assessed by Spearman’s correlation coefficient. *p* < .05 was defined as statistically significant.

## 3 Results

### 3.1 Trends in publications and citations

**FIGURE 2:**
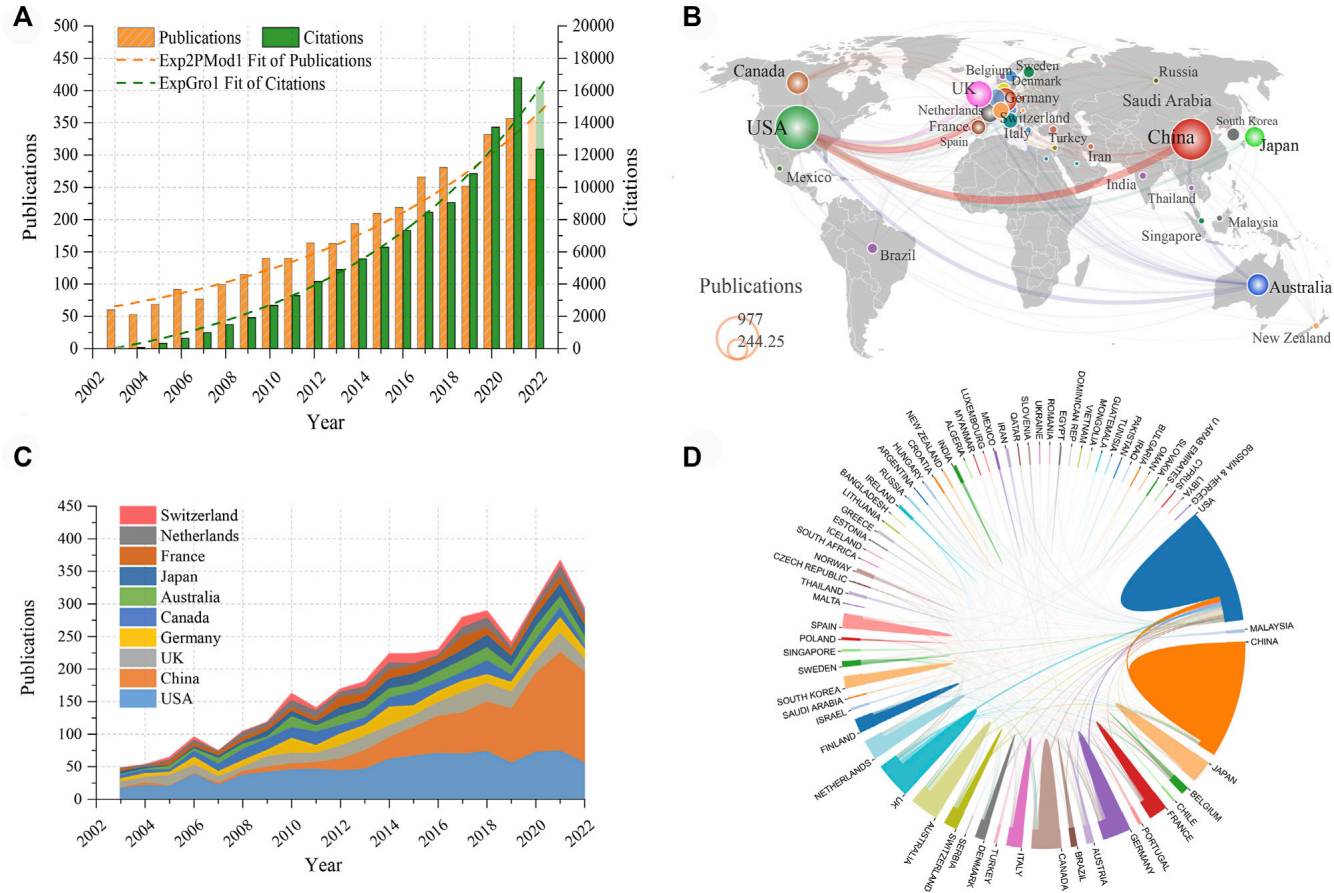
**(A)** Trends in annual citations and publications from 2003 to 2022. **(B)** World map of the number of publications from each country. The size of the circles represents the number of publications, and the thickness of the lines represents the intensity of cooperation between two countries. **(C)** Trends in annual publication count in the top 10 productive countries. **(D)** Network diagram of cooperation among countries.

A total of 3,545 publications were finally included in the analysis, including 3,006 (84.8%) original articles and 539 (15.2%) reviews. Almost all were English publications (3,485, 98.3%), followed by German (26, .7%) and French (8, .2%). The detailed distribution of annual publications for subchondral bone research was shown in Figure 2A. An exponential growth trend in the number of annual publications and citations related to subchondral bone can be observed. In terms of citation counts, the cumulative total number of citations (TC) for all publications was 111,015 (87,040 after removing self-citations), the average number of citations (AC) per publication was 31.32, and the HI was 130.

### 3.2 Global research status and knowledge landscape

#### 3.2.1 Analysis of countries

The majority of the works were published by researchers from Europe, North America, and East Asia, as can be seen from the global map of national contributions (Figure 2B). Specifically, as shown in Table 1, the US had the most publications in this field with 980 papers, followed by China (862) and the UK (364), while the other countries published less than 300 papers. The USA also ranked first in terms of TC and HI, followed by the UK. In addition, the correlation analysis showed a high positive correlation between the total number of publications (TP) and GDP (*r* = .948, *p* < .001). As shown in Figure 2C, the US dominated the number of papers in this field until 2018, while China had tremendous development beginning in 2012 and eventually overtook the US after 2018. The international cooperation among the different countries was illustrated in Figure 2D. The thickness of the lines between the two countries indicates the strength of cooperation. It can be seen that the US exhibited the strongest collaboration with China, Germany, and the United Kingdom.

**TABLE 1:** Top 10 countries and institutions with the most publications.

Rank	Country/Institution	TP	TC	AC	HI
	Country				
1	United States	980	41,150	41.99	98
2	China	862	15,893	18.44	54
3	United Kingdom	364	16,203	44.51	67
4	Germany	297	9,819	33.06	56
5	Canada	254	12,494	49.19	56
6	Australia	240	9,162	38.18	51
7	Japan	212	4,955	23.37	39
8	France	183	9,588	52.39	53
9	Netherlands	156	8,983	57.58	43
10	Switzerland	138	6,007	43.53	36
	Institution				
1	University of California System	125	5,210	41.68	41
2	Université de Montréal	107	8,760	81.87	46
3	Harvard University	99	2,929	29.59	31
4	Udice French Research Universities	83	4,946	59.59	30
5	University of London	81	3,972	49.04	28
6	Boston University	79	3,805	48.16	35
7	APHP	78	5,078	65.10	33
8	Shanghai Jiao Tong University	76	2,064	27.16	21
9	University of Sydney	71	2,433	34.27	26
10	Inserm	60	3,111	51.85	26

TP, total number of publications; TC, total number of citations; AC, average number of citations; HI, hirsch index; APHP, assistance publique hopitaux Paris; Inserm, Institut national de la santé et de la recherche médicale.

#### 3.2.2 Analysis of institutions

**FIGURE 3:**
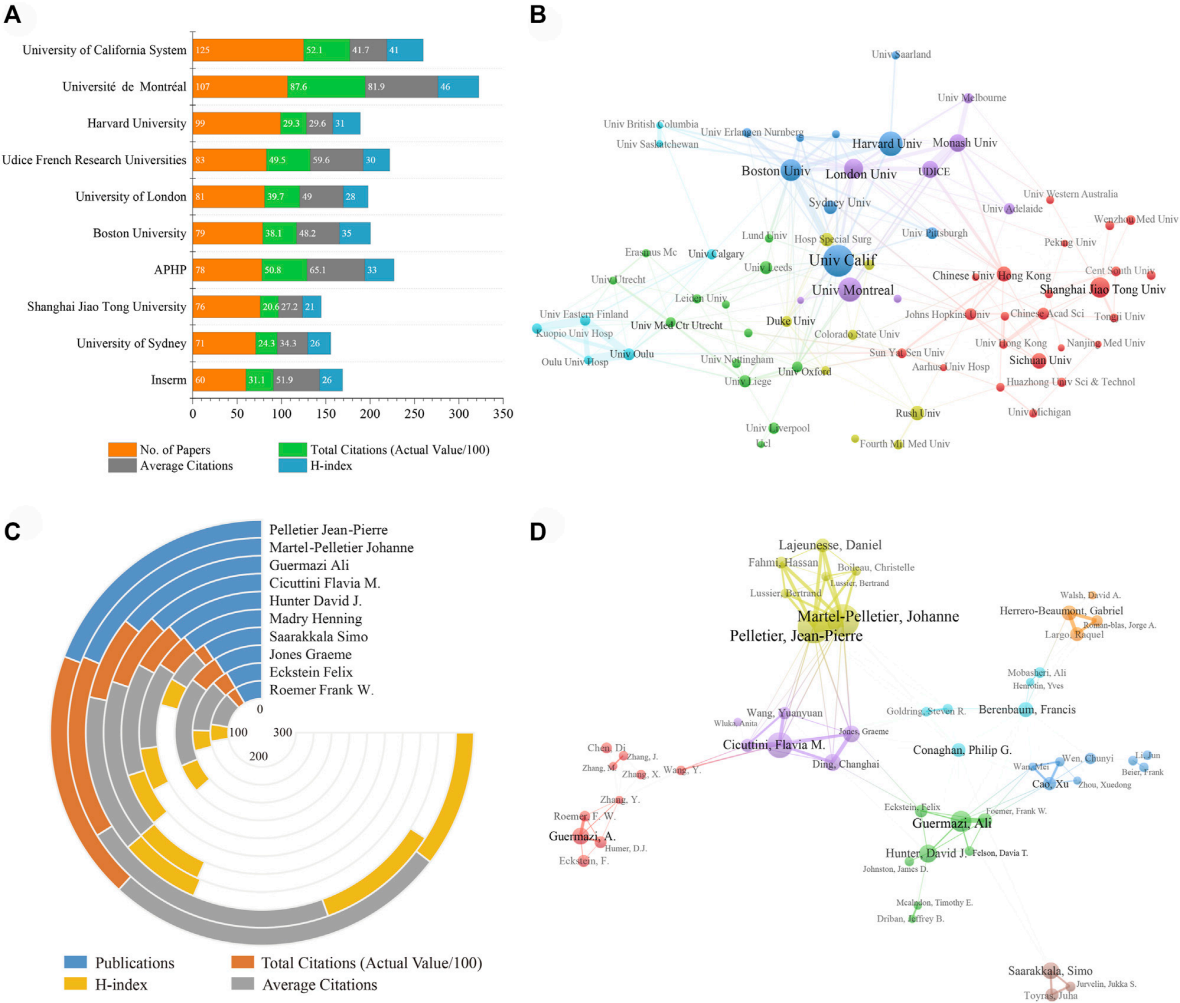
**(A)** The publication counts, citations, and H-index of the top 10 prolific institutions. **(B)** Cooperation network among institutions. **(C)** The publication counts, citations, and H-index of the top 10 most productive authors. **(D)** Visualization diagram of co-authorship analysis for authors. The size of the circles represents the number of publications, and the thickness of the connecting lines represents the strength of collaboration between two institutions/authors.

It was estimated that more than 2,900 institutions contributed to this field. Figure 3A and Table 1 showed the top 10 most productive institutions. Three of these are from the United States, three are from France, and the others are from Australia, China, the United Kingdom, and Canada. Specifically, the University of California System ranked first with 125 articles, while Université de Montréal had the highest TC (8,760), AC (81.87), and HI (46). 68 institutions published no less than 20 articles. Figure 3B illustrated the close and complex cooperation among different institutions. It is clear that the University of California System and Boston University were the centers of the most collaborations, indicating the strong influence of these institutions.

#### 3.2.3 Analysis of authors

In terms of the top 10 prolific authors (Table 2; Figure 3C), Pelletier Jean-Pierre from the Université de Montréal ranked first with 72 publications, followed by Martel-pelletier Johanne (70) and Guermazi Ali (53). In addition, Pelletier Jean-Pierre was also the author with the highest TC, AC, and HI. A visualization of the author’s co-authorship analysis was generated by VOSviewer software (Figure 3D). It can be seen that Pelletier Jean-Pierre had the most collaborations centered on him, indicating his strong influence in subchondral bone research. Pelletier Jean-Pierre and Martel-pelletier Johanne have the highest co-authorship intensity (total link strength = 136) with 14 articles, followed by Cicuttini Flavia M. (total link strength = 75) with 11 articles.

**TABLE 2:** Top 10 most prolific authors on subchondral bone research in osteoarthritis.

Rank	Author	Affiliation of the author	TP	TC	AC	HI
1	Pelletier Jean-Pierre	Université de Montréal, Canada	72	7,147	99.26	39
2	Martel-Pelletier Johanne	Université de Montréal, Canada	70	5,709	81.56	39
3	Guermazi Ali	Boston University, United States	53	2,875	54.25	28
4	Cicuttini Flavia M	Monash University, Australia	46	2,829	61.50	27
5	Hunter David J	University of Sydney, Australia	43	2,085	48.49	23
6	Madry Henning	Saarland University, Germany	41	1,769	43.15	21
7	Saarakkala Simo	University of Oulu, Finland	35	697	19.91	16
8	Jones Graeme	University of Tasmania, Australia	34	2,110	62.06	20
9	Eckstein Felix	Paracelsus Medical University, Austria	32	1,263	39.47	20
10	Roemer Frank W	Boston University, United States	32	1,451	45.34	18

TP, total number of publications; TC, total number of citations; AC, average number of citations; HI, hirsch index.

#### 3.2.4 Analysis of journals and grants

In total, 1,071 (30.2%) articles were published in the top 10 most prolific journals. As shown in Table 3, Osteoarthritis and Cartilage (431, 12.2%) had the highest output, followed by Journal of Orthopaedic Research (116, 4.39%) and Annals of the Rheumatic Diseases (123, 3.3%). Half of the top 10 academic journals belong to Q1. Annals of the Rheumatic Diseases has the highest IF, followed by Arthritis and Rheumatology. In terms of publishers, most of these active journals were from Europe and North America. In terms of grants, NSFC funded the most articles (474, 13.4%), followed by HHS (445, 12.6%), NIH (438, 12.4%), NIAMS (282, 8.0%), and European Commission (162, 4.6%), while other grants funded less than 100 articles, as shown in Table 4.

**TABLE 3:** The top 10 journals contributing to publications.

Rank	Journal	TP	TC	AC	HI	IF	JQ
1	Osteoarthritis and Cartilage	431	21,602	50.12	75	7.507	Q1
2	Journal of Orthopaedic Research	116	2,468	21.28	27	3.102	Q2
3	Arthritis Research and Therapy	96	4,887	50.91	41	5.606	Q1
4	Bone	79	3,072	38.89	31	4.626	Q2
5	Bmc Musculoskeletal Disorders	70	1,209	17.27	19	2.562	Q4
6	Arthritis and Rheumatology^a^	61	5,537	90.77	45	15.483	Q1
7	Scientific Reports	61	968	15.87	18	4.996	Q2
8	American Journal of Sports Medicine	56	1,840	32.86	24	7.010	Q1
9	Annals of the Rheumatic Diseases	51	3,661	71.78	35	27.973	Q1
10	International Journal of Molecular Sciences	50	1,215	24.30	15	6.208	Q2

^a^ Arthritis and Rheumatism relaunched as Arthritis and Rheumatology after 2015. The data from these journals were merged. TP, total number of publications; TC, total number of citations; AC, average number of citations; HI, hirsch index; IF, impact factor; JQ, journal quartiles.

**TABLE 4:** Top 10 funds with the most publications on subchondral bone research in osteoarthritis.

Rank	Funds	TP	%	TC	AC	HI
1	National Natural Science Foundation of China (NSFC)	474	13.4	8,220	17.34	45
2	United States Department of Health Human Services (HHS-US)	445	12.6	21,023	47.24	74
3	National Institutes of Health (NIH-US)	438	12.4	20,852	47.61	74
4	National Institute of Arthritis Musculoskeletal Skin Diseases (NIAMS)	282	8.0	16,112	57.13	68
5	European Commission	162	4.6	6,147	37.94	42
6	UK Research Innovation (UKRI)	94	2.7	4,019	42.76	36
7	National Health and Medical Research Council (NHMRC-Australia)	91	2.6	2,952	32.44	30
8	Ministry of Education Culture Sports Science and Technology (MEXT-Japan)	86	2.4	2,032	23.63	26
9	Canadian Institutes of Health Research (CIHR)	83	2.3	2,418	29.13	29
10	National Institute on Aging (NIA)	75	2.1	6,404	85.39	42

TP, total number of publications; TC, total number of citations; AC, average number of citations; HI, hirsch index.

### 3.3 Analysis of highly cited literature

**FIGURE 4:**
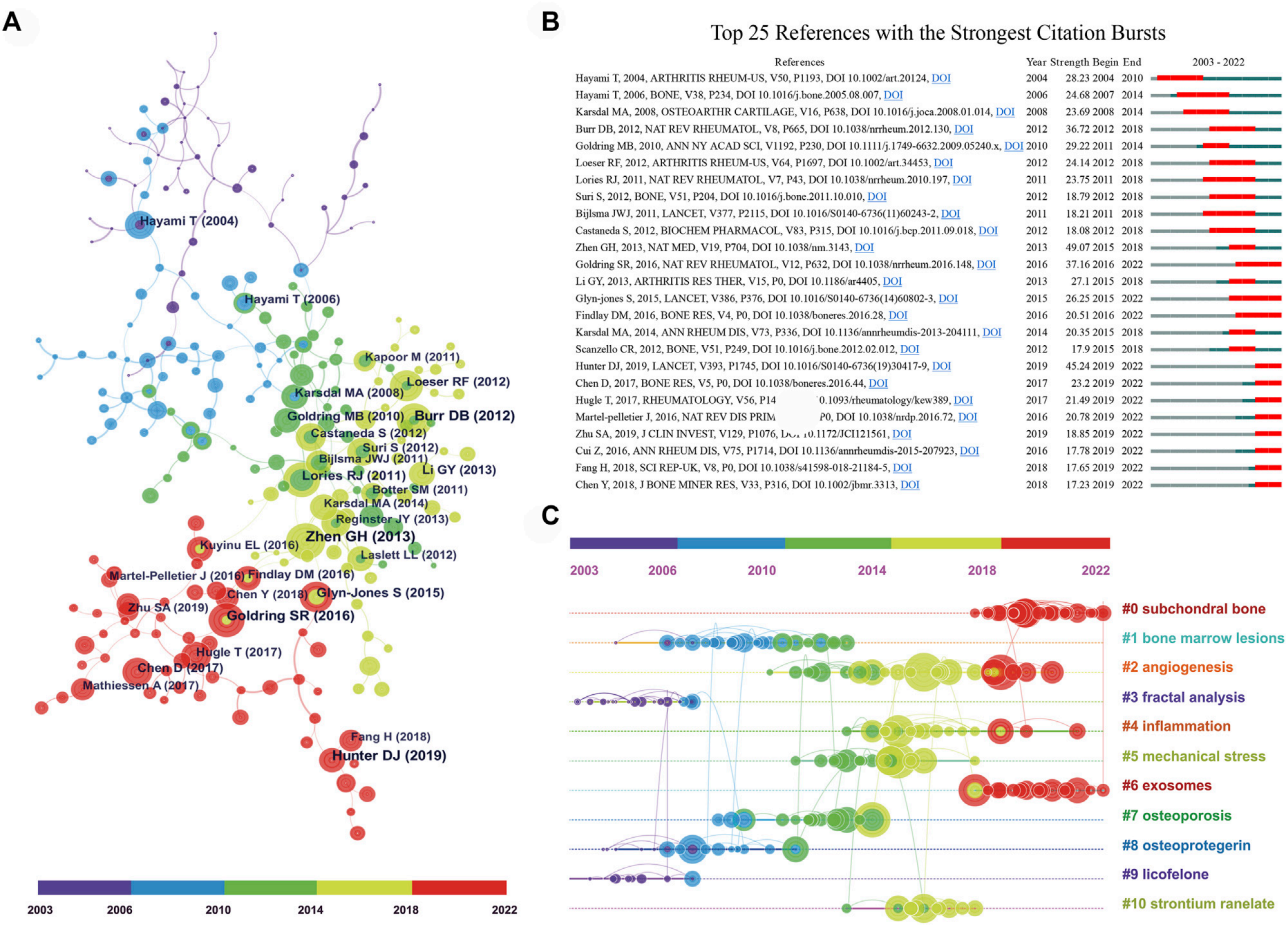
**(A)** The co-cited reference knowledge map. The node size represents the number of co-citations. **(B)** The top 25 references with the strongest citation bursts. The blue line indicates the time interval and the red part represents the period of the reference burst. **(C)** The timeline graph of co-cited reference clusters.

Table 5 listed the top 10 most cited papers in subchondral bone research with citations ranging from 391 to 1,570. Of these, four were original articles with TC of 2,132, and six were reviews with TC of 4,969, which indicated that review articles tend to have higher citations. Specifically, the top three most cited papers were all reviews. With 1,570 citations, the review produced by Kapoor et al. (2011) attracted the greatest attention, followed by the work from (Berenbaum et al., 2013) with 951 citations. The most cited original article ranked fourth with 609 citations. The knowledge graph of highly co-cited references was shown in Figure 4A. Figure 4B specifically presented the top 25 references with the strongest citation bursts. The citation burst analysis could identify references with a significant increase in citations during a short period of time and could also reflect the focus of research in a given period (Kleinberg, 2003). Figure 4B showed that the reference with the strongest citation burst value was written by Zhen et al. (2013), followed by Hunter and Bierma-Zeinstra (2019).

**TABLE 5:** Top 10 high-cited articles on subchondral bone research in osteoarthritis.

Rank	First author	Year	Article title	Journal	TC
1	Kapoor, M	2011	Role of proinflammatory cytokines in the pathophysiology of osteoarthritis	Nature Reviews Rheumatology	1570
2	Berenbaum, F	2013	Osteoarthritis as an inflammatory disease (osteoarthritis is not osteoarthrosis!)	Osteoarthritis and Cartilage	951
3	Dieppe, PA	2005	Pathogenesis and management of pain in osteoarthritis	Lancet	756
4	Zhen, GH	2013	Inhibition of TGF-beta signaling in mesenchymal stem cells of subchondral bone attenuates osteoarthritis	Nature Medicine	609
5	Goldring, MB	2010	Articular cartilage and subchondral bone in the pathogenesis of osteoarthritis	Skeletal Biology and Medicine	538
6	Chen, D	2017	Osteoarthritis: toward a comprehensive understanding of pathological mechanism	Bone Research	529
7	Hayami, T	2006	Characterization of articular cartilage and subchondral bone changes in the rat anterior cruciate ligament transection and meniscectomized models of osteoarthritis	Bone	515
8	Burr, DB	2012	Bone remodelling in osteoarthritis	Nature Reviews Rheumatology	499
9	Hayami, T	2004	The role of subchondral bone remodeling in osteoarthritis - Reduction of cartilage degeneration and prevention of osteophyte formation by alendronate in the rat anterior cruciate ligament transection model	Arthritis and Rheumatism^a^	470
10	Li, GY	2013	Subchondral bone in osteoarthritis: insight into risk factors and microstructural changes	Arthritis Research and Therapy	391

^a^ Arthritis and Rheumatism relaunched as Arthritis and Rheumatology after 2015. TC, total number of citations.

### 3.4 Overview of research hotspots and frontiers

#### 3.4.1 Cluster analysis of co-cited references

The references in the co-cited network (Figure 4A) were classified into 11 different clusters by cluster analysis, as shown in the timeline view (Figure 4C). The references in the same cluster were arranged in one timeline by publication time. These clusters were labeled by extricating terms from the titles of cited papers using the log-likelihood ratio algorithm (Wen et al., 2022b; You et al., 2022). As can be seen in Figure 4C, “subchondral bone” was the largest cluster (#0), followed by “bone marrow lesions” (#1) and “angiogenesis” (#2). In addition, the evolutionary characteristics of each cluster can be seen at a glance in this timeline view. We can see that clusters of subchondral bone (#0), angiogenesis (#2), inflammation (#4), and exosomes (#6) have been hot topics in recent years.

#### 3.4.2 Analysis of keywords

**FIGURE 5:**
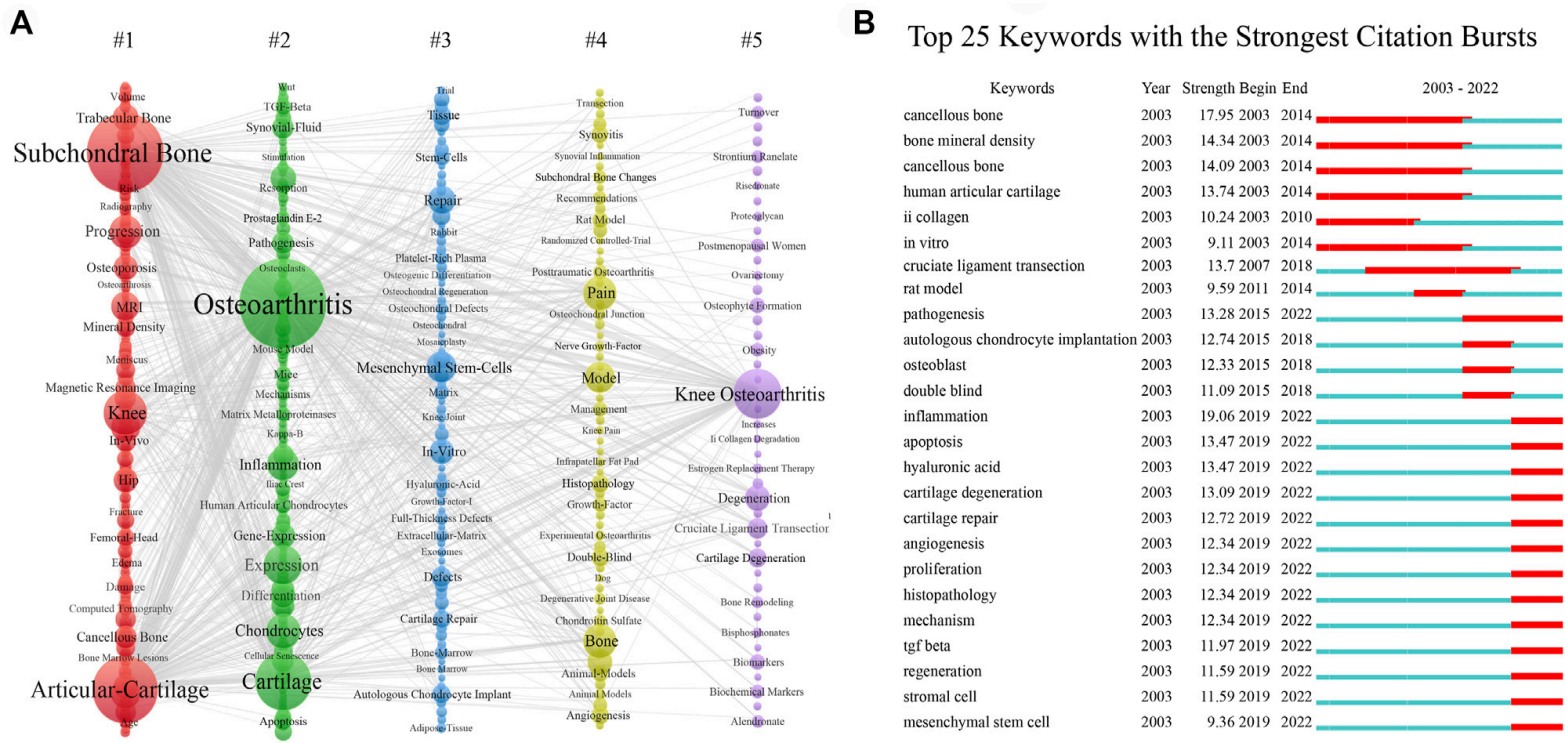
**(A)** The co-occurrence view of the keywords, in which each column represents a cluster of studies generated by VOSviewer. The size of the node is proportional to the number of keyword occurrences; the thickness of the line is proportional to the number of keyword co-occurrences. **(B)** The top 20 keywords with the strongest citation bursts.

Figure 5A showed a visualization of keywords that co-occurred more than 10 times in the subchondral bone research. In total, 493 keywords were grouped into five clusters. Based on the keywords in the groups, they can be summarized as follows: #1 subchondral bone, #2 pathogenesis, #3 therapeutics, #4 pain, and #5 degeneration. The top 25 keywords with the strongest citation burst were listed in Figure 5B, where the burst period lasted until the present day were pathogenesis, inflammation, apoptosis, cartilage degeneration/repair, angiogenesis, TGF beta, regeneration, stromal cell, mesenchymal stem cell.

## 4 Discussion

This study provided a bibliometric analysis of publications on subchondral bone research in OA, which can help beginners intuitively and systematically understand the development process and trends in this field. We found that the TP and TC in subchondral bone research have been increasing exponentially over the past 20 years (Figure 2A). Accordingly, we believe that subchondral bone has attracted substantial and consistently growing attention from scholars and that it is an important research direction for OA research. This trend may be related to the increasing global incidence and prevalence of OA due to the aging and obesity of the population (Chen et al., 2017; Hunter and Bierma-Zeinstra, 2019; Kloppenburg and Berenbaum, 2020).

This study found that the US ranked first in the world in terms of TP, TC, and HI, indicating its dominant influence in subchondral bone research (Figure 2B; Table 1). Of note, despite being second in TP, China had fewer AC than any other country, probably because most of the publications from China were published in recent years and have not yet accumulated enough citations (Figure 2C). In terms of institutions, the University of California System contributed the most, while Université de Montréal had the highest TC, AC, and HI, indicating their leadership in subchondral bone research (Table 1; Figure 3A). Co-authorship analysis can establish similar relationships between individuals or institutions by evaluating the number of co-authored publications to understand the status of collaborations among them (Wu et al., 2021; Grover and Gupta, 2022). Figures 2D, 3B demonstrated that collaborations among institutions from North America, East Asia, and Europe were stronger, while collaborations among other institutions were substantially weaker. National and institutional output and cooperation may be influenced by economic power. This study found a significant positive correlation between output and GDP. Countries with high GDP would be likely to invest heavily in scientific investigations and produce a large number of senior researchers (Kim et al., 2016; Wu et al., 2021). It is noteworthy that publications from China have experienced rapid growth since 2012, which is inextricably linked to economic development and funding support (Table 4). Finally, greater mutual collaboration and communication between institutions is encouraged to promote OA research.

Pelletier Jean-Pierre (Université de Montréal, Canada) had particularly attractive data (Table 2; Figure 3C, 3D), indicating his powerful academic influence in the field. His team has been working on a series of studies on the pathogenesis, risk factors, biomarkers, and treatment of OA. In a recent study, they identified single nucleotide polymorphism genes and mitochondrial DNA haplogroups as biomarkers for early prediction of the progression of knee OA through machine learning (Bonakdari et al., 2022; Martel-Pelletier and Pelletier, 2022). Highly cited literature can be considered the most valuable and influential research in the field, so new researchers can read these papers before further research (Table 5; Figure 4). For example, the article “Role of proinflammatory cytokines in the pathophysiology of osteoarthritis” by Kapoor et al. (2011). was ranked first with 1,570 citations. This article reviewed the role of pro-inflammatory cytokines (IL-1β, TNF, IL-6, IL-15, IL-17, IL-18, IL-21, IL-8, and leukemia inhibitory factor) in the pathophysiology of OA and explored the potential of anti-cytokine therapy for OA (Kapoor et al., 2011). Berenbaum et al (2013) suggested that subchondral bone may be the source of inflammatory mediators of pain and deep cartilage degradation processes in OA. Furthermore, the article by Zhen et al. (2013) had the strongest citation burst value of 49.07, which means that this study was widely cited in the short term. His study showed that high levels of active TGF-β1 in subchondral bone appear to trigger pathological changes in OA. High concentrations of TGF-β1 could induce the formation of nestin-positive mesenchymal stem cell (MSC) clusters, leading to the formation of bone marrow-like islets and accompanied by angiogenesis. In contrast, suppressing TGF-β activity in subchondral bone could attenuate articular cartilage degeneration. Moreover, the article by Hunter and Bierma-Zeinstra (2019) detailing the updated information on the pathogenesis, diagnosis, treatment, and frontiers of OA was the paper with the strongest citation burst in recent years. All of the above literature has had a profound impact on OA research.

In this study, keyword analysis and literature analysis were used to obtain research hotspots and predict Frontier trends. It is reported that research themes in the field could be reflected by cluster analysis of co-cited references and cluster analysis of co-occurrence keywords (Chen et al., 2012; Zhang X. et al., 2021). The evolution of research hotspots could be revealed by the timeline view of clusters (Chen et al., 2012). In this study, the co-cited references were classified into 11 clusters by CiteSpace software, of which four clusters subchondral bone (#0), angiogenesis (#2), inflammation (#4), and exosomes (#6) were hot topics in recent years (Figure 4C). In addition, the burst detection algorithm can capture the sharp increase in keyword popularity over a specific period, which can be an effective way to identify topics discussed actively during this period (Kleinberg, 2003), as shown in Figure 5B. It demonstrated that hot keywords have focused on pathomechanisms and therapeutics in recent years.

Subchondral bone provides mechanical and nutritional support to cartilage and plays a crucial role in the pathogenesis of OA. Alterations in the cellular and extracellular components of the subchondral bone microenvironment can cause abnormalities in subchondral bone remodeling, angiogenesis, and sensory innervation, thereby leading to cartilage destruction and pain (Hu W. et al., 2021; Hu Y. et al., 2021). In addition, subchondral bone can affect cartilage degeneration through mechanical alterations or paracrine-mediated bone-chondral crosstalk (Zhen et al., 2013; Lin et al., 2019; Ni et al., 2020). When subchondral bone is injured, such as subchondral bone cysts or subchondral bone marrow lesions (BML), the integrity of the osteochondral unit is disrupted, crosstalk between cartilage and subchondral bone is increased, and subchondral bone remodeling is aberrant (Goldring and Goldring, 2016; Muratovic et al., 2016). Osteoclasts, monocytes, osteoblasts, and osteocytes are the main cells involved in subchondral bone remodeling (Sims and Martin, 2020). In addition, bioactive factors in the bone extracellular matrix are also involved in this process, such as TGFβ, which could cause the recruitment of MSCs and enhance bone formation (Zhen et al., 2013). Aggressive subchondral H-type angiogenesis and abnormal sensory innervation are also key processes in subchondral bone remodeling. Netrin-1 secreted by osteoblasts could induce sensory nerve growth during subchondral bone remodeling (Zhu et al., 2019). Platelet-derived growth factor-BB (PDGF-BB) over-secreted by preosteoclasts could enhance subchondral angiogenesis (Xie et al., 2014; Su et al., 2020). Vascular endothelial growth factor (VEGF) derived from osteoblasts and mast chondrocytes could also stimulate angiogenesis (Hu and Olsen, 2016). The above pathological processes individually or synergistically contribute to OA progression.

Although there are no treatments designed specifically for subchondral BML, several drugs have shown great therapeutic potential in animal models given the above pathogenesis, as subchondral bone changes precede cartilage degeneration. 1) Restoring abnormal subchondral bone remodeling using antiresorptive agents has been evaluated in many trials. Evidence demonstrated that osteoprotegerin, bisphosphonates, cathepsin K inhibitors, and strontium ranelate have protective effects on subchondral bone and cartilage in animal models or clinical trials (Sagar et al., 2014; Han et al., 2017; Conaghan et al., 2020; Hayes et al., 2020). However, recent meta-analyses showed that bisphosphonates do not improve pain, BML, or function in patients with knee OA (Vaysbrot et al., 2018; Zhang X. et al., 2022). Moreover, TGF-β1 antibodies could block the phosphorylation of Smad2/3 in preosteoclasts in OA models, thereby reducing their subchondral localization and improving bone parameters and cartilage architecture (Zhen et al., 2013). 2) Bevacizumab (VEGF blocking antibody) and defactinib (focal adhesion kinase inhibitor) can reduce subchondral H-vessel formation in a mouse model of OA, thereby inhibiting chondrocyte hypertrophy and delaying OA progression (Lu et al., 2018; Hu et al., 2020). Inhibition of Netrin-1 in subchondral osteoblasts could attenuate sensory innervation and relieve pain (Zhu et al., 2019). Of note, more clinical trials are needed for these therapeutic agents and potential therapeutic targets. 3) The value of regenerative therapies has been confirmed by many studies. MSCs play a key role in subchondral bone remodeling and osteochondral homeostasis (Zhu et al., 2021). In a series of 15-year clinical follow-up trials, (Hernigou et al., 2021a; Hernigou et al., 2021b) demonstrated that treatment with bone marrow MSCs was effective in reducing subchondral BML and could delay joint replacement in OA patients for more than 10 years. In addition, stem cell-derived exosomes may protect joints from damage by promoting cartilage repair and mediating subchondral bone remodeling (Ni et al., 2020). However, investigations into MSC-derived exosomes are just beginning, and there are still many unanswered questions.

## 5 Limitations

To our knowledge, this work is the first comprehensive bibliometric analysis of publications related to subchondral bone in OA. However, this study also has several limitations inherent in bibliometrics. Firstly, the data was only sourced from the WoSCC database, which may have missed some relevant studies from other databases. However, WoSCC is the most widely used and sufficiently large database for bibliometric study, as indicated in earlier studies (Zhang Y. et al., 2021; Wu et al., 2021). Secondly, the shortcoming is that this study did not perform journal dual-map overlay analysis because it can reflect the citation relationship between journals and further show the change in the research area. Finally, recent high-quality works may not have received enough citations, which could lead to an underestimation of their influence. Although the WoSCC database is continuously updated and citation-specific parameters are subject to temporal changes, the effect of time on citation trends is limited. Therefore, this work can shed light on the general status and trends of subchondral bone research in OA.

## 6 Conclusion

This study found that publications on subchondral bone in OA have experienced an exponential increase. The pathological mechanisms of cellular and molecular interactions in the subchondral bone microenvironment in the development of OA, as well as the exploitation of targeted therapeutic agents against the altered subchondral bone microenvironment, would be current hot topics and future research trends.

## Data Availability

The original contributions presented in the study are included in the article/supplementary material, further inquiries can be directed to the corresponding authors.

## References

[B1] Bannuru R. R. Osani M. C. Vaysbrot E. E. Arden N. K. Bennell K. Bierma-Zeinstra S. M. A. (2019). OARSI guidelines for the non-surgical management of knee, hip, and polyarticular osteoarthritis. Osteoarthr. Cartil. 27, 1578–1589. 10.1016/j.joca.2019.06.011 31278997

[B2] Berenbaum F. (2013). Osteoarthritis as an inflammatory disease (osteoarthritis is not osteoarthrosis!). Osteoarthr. Cartil. 21, 16–21. 10.1016/j.joca.2012.11.012 23194896

[B3] Bonakdari H. Pelletier J. P. Blanco F. J. Rego-Perez I. Duran-Sotuela A. Aitken D. (2022). Single nucleotide polymorphism genes and mitochondrial DNA haplogroups as biomarkers for early prediction of knee osteoarthritis structural progressors: Use of supervised machine learning classifiers. BMC Med. 20, 316. 10.1186/s12916-022-02491-1 36089590 PMC9465912

[B4] Chen C. Hu Z. Liu S. Tseng H. (2012). Emerging trends in regenerative medicine: A scientometric analysis in CiteSpace. Expert Opin. Biol. Ther. 12, 593–608. 10.1517/14712598.2012.674507 22443895

[B5] Chen D. Shen J. Zhao W. Wang T. Han L. Hamilton J. L. (2017). Osteoarthritis: Toward a comprehensive understanding of pathological mechanism. Bone Res. 5, 16044. 10.1038/boneres.2016.44 28149655 PMC5240031

[B6] Conaghan P. G. Bowes M. A. Kingsbury S. R. Brett A. Guillard G. Rizoska B. (2020). Disease-Modifying effects of a novel cathepsin K inhibitor in osteoarthritis: A randomized controlled trial. Ann. Intern Med. 172, 86–95. 10.7326/M19-0675 31887743

[B7] Dieppe P. A. Lohmander L. S. (2005). Pathogenesis and management of pain in osteoarthritis. Lancet (London, Engl. 365, 965–973. 10.1016/s0140-6736(05)71086-2 15766999

[B8] Goldring S. R. Goldring M. B. (2016). Changes in the osteochondral unit during osteoarthritis: Structure, function and cartilage-bone crosstalk. Nat. Rev. Rheumatol. 12, 632–644. 10.1038/nrrheum.2016.148 27652499

[B9] Grover S. Gupta B. M. (2022). Global research on obsessive compulsive disorder and related disorders: A scientometric assessment of global research during 2002-2021. Asian J. Psychiatr. 72, 103146. 10.1016/j.ajp.2022.103146 35537321

[B10] Han W. Fan S. Bai X. Ding C. (2017). Strontium ranelate, a promising disease modifying osteoarthritis drug. Expert Opin. investigational drugs 26, 375–380. 10.1080/13543784.2017.1283403 28092725

[B11] Hayes K. N. Giannakeas V. Wong A. K. O. (2020). Bisphosphonate use is protective of radiographic knee osteoarthritis progression among those with low disease severity and being non-overweight: Data from the osteoarthritis initiative. J. bone mineral Res. official J. Am. Soc. Bone Mineral Res. 35, 2318–2326. 10.1002/jbmr.4133 32662919

[B12] Hernigou P. Bouthors C. Bastard C. Flouzat Lachaniette C. H. Rouard H. Dubory A. (2021a). Subchondral bone or intra-articular injection of bone marrow concentrate mesenchymal stem cells in bilateral knee osteoarthritis: What better postpone knee arthroplasty at fifteen years? A randomized study. Int. Orthop. 45, 391–399. 10.1007/s00264-020-04687-7 32617651

[B13] Hernigou P. Delambre J. Quiennec S. Poignard A. (2021b). Human bone marrow mesenchymal stem cell injection in subchondral lesions of knee osteoarthritis: A prospective randomized study versus contralateral arthroplasty at a mean fifteen year follow-up. Int. Orthop. 45, 365–373. 10.1007/s00264-020-04571-4 32322943

[B14] Hu K. Olsen B. R. (2016). Osteoblast-derived VEGF regulates osteoblast differentiation and bone formation during bone repair. J. Clin. investigation 126, 509–526. 10.1172/JCI82585 PMC4731163 26731472

[B15] Hu W. Chen Y. Dou C. Dong S. (2021a). Microenvironment in subchondral bone: Predominant regulator for the treatment of osteoarthritis. Ann. rheumatic Dis. 80, 413–422. 10.1136/annrheumdis-2020-218089 PMC7958096 33158879

[B16] Hu W. S. Zhang Q. Li S. H. Ai S. C. Wu Q. F. (2022). Ten hotspot MicroRNAs and their potential targets of chondrocytes were revealed in osteoarthritis based on bibliometric analysis. J. Healthc. Eng. 2022, 8229148. 10.1155/2022/8229148 35437466 PMC9013302

[B17] Hu Y. Chen X. Wang S. Jing Y. Su J. (2021b). Subchondral bone microenvironment in osteoarthritis and pain. Bone Res. 9, 20. 10.1038/s41413-021-00147-z 33731688 PMC7969608

[B18] Hu Y. Wu H. Xu T. Wang Y. Qin H. Yao Z. (2020). Defactinib attenuates osteoarthritis by inhibiting positive feedback loop between H-type vessels and MSCs in subchondral bone. J. Orthop. Transl. 24, 12–22. 10.1016/j.jot.2020.04.008 PMC7261948 32518750

[B19] Hügle T. Geurts J. (2017). What drives osteoarthritis?-synovial versus subchondral bone pathology. Rheumatol. Oxf. Engl. 56, 1461–1471. 10.1093/rheumatology/kew389 28003493

[B20] Hunter D. J. Bierma-Zeinstra S. (2019). Osteoarthritis. Lancet (London, Engl. 393, 1745–1759. 10.1016/S0140-6736(19)30417-9 31034380

[B21] Kapoor M. Martel-Pelletier J. Lajeunesse D. Pelletier J. P. Fahmi H. (2011). Role of proinflammatory cytokines in the pathophysiology of osteoarthritis. Nat. Rev. Rheumatol. 7, 33–42. 10.1038/nrrheum.2010.196 21119608

[B22] Kim H. J. Yoon D. Y. Kim E. S. Lee K. Bae J. S. Lee J. H. (2016). The 100 most-cited articles in neuroimaging: A bibliometric analysis. Neuroimage 139, 149–156. 10.1016/j.neuroimage.2016.06.029 27327516

[B23] Kleinberg J. (2003). Bursty and hierarchical structure in streams. Data Min. Knowl. Discov. 7, 373–397. 10.1023/A:1024940629314

[B24] Kloppenburg M. Berenbaum F. (2020). Osteoarthritis year in review 2019: Epidemiology and therapy. Osteoarthr. Cartil. 28, 242–248. 10.1016/j.joca.2020.01.002 31945457

[B25] Kroon F. P. B. Carmona L. Schoones J. W. Kloppenburg M. (2018). Efficacy and safety of non-pharmacological, pharmacological and surgical treatment for hand osteoarthritis: A systematic literature review informing the 2018 update of the EULAR recommendations for the management of hand osteoarthritis. RMD open 4, e000734. 10.1136/rmdopen-2018-000734 30402266 PMC6203105

[B26] Lin C. Liu L. Zeng C. Cui Z. K. Chen Y. Lai P. (2019). Activation of mTORC1 in subchondral bone preosteoblasts promotes osteoarthritis by stimulating bone sclerosis and secretion of CXCL12. Bone Res. 7, 5. 10.1038/s41413-018-0041-8 30792936 PMC6381187

[B27] Lu J. Zhang H. Cai D. Zeng C. Lai P. Shao Y. (2018). Positive-Feedback regulation of subchondral H-type vessel formation by chondrocyte promotes osteoarthritis development in mice. J. bone mineral Res. official J. Am. Soc. Bone Mineral Res. 33, 909–920. 10.1002/jbmr.3388 29329496

[B28] Martel-Pelletier J. Pelletier J. P. (2022). Is there a mitochondrial DNA haplogroup connection between osteoarthritis and elite athletes? A narrative review. RMD open 8, e002602. 10.1136/rmdopen-2022-002602 36113964 PMC9486370

[B29] Muratovic D. Cicuttini F. Wluka A. Findlay D. Wang Y. Otto S. (2016). Bone marrow lesions detected by specific combination of MRI sequences are associated with severity of osteochondral degeneration. Arthritis Res. Ther. 18, 54. 10.1186/s13075-016-0953-x 26912313 PMC4766616

[B30] Ni Z. Zhou S. Li S. Kuang L. Chen H. Luo X. (2020). Exosomes: Roles and therapeutic potential in osteoarthritis. Bone Res. 8, 25. 10.1038/s41413-020-0100-9 32596023 PMC7305215

[B31] Sagar D. R. Ashraf S. Xu L. Burston J. J. Menhinick M. R. Poulter C. L. (2014). Osteoprotegerin reduces the development of pain behaviour and joint pathology in a model of osteoarthritis. Ann. rheumatic Dis. 73, 1558–1565. 10.1136/annrheumdis-2013-203260 PMC4112443 23723320

[B32] Sims N. A. Martin T. J. (2020). Osteoclasts provide coupling signals to osteoblast lineage cells through multiple mechanisms. Annu. Rev. Physiol. 82, 507–529. 10.1146/annurev-physiol-021119-034425 31553686

[B33] Song W. Ma T. Cheng Q. Wen P. Wu J. Hao L. (2022). Global research status and trends in venous thromboembolism after hip or knee arthroplasty from 1990 to 2021: A bibliometric analysis. Front. Med. (Lausanne) 9, 837163. 10.3389/fmed.2022.837163 35462997 PMC9021752

[B34] Su W. Liu G. Liu X. Zhou Y. Sun Q. Zhen G. (2020). Angiogenesis stimulated by elevated PDGF-BB in subchondral bone contributes to osteoarthritis development. JCI insight 5, e135446. 10.1172/jci.insight.135446 32208385 PMC7205438

[B35] Vaysbrot E. E. Osani M. C. Musetti M. C. McAlindon T. E. Bannuru R. R. (2018). Are bisphosphonates efficacious in knee osteoarthritis? A meta-analysis of randomized controlled trials. Osteoarthr. Cartil. 26, 154–164. 10.1016/j.joca.2017.11.013 29222056

[B36] Wen P. Luo P. Zhang B. Wang Y. Hao L. Wang J. (2022a). Hotspots and future directions in rheumatoid arthritis-related cardiovascular disease: A scientometric and visualization study from 2001 to 2021 based on Web of science. Front. Med. (Lausanne) 9, 931626. 10.3389/fmed.2022.931626 35966862 PMC9372309

[B37] Wen P. Luo P. Zhang B. Zhang Y. (2022b). Mapping knowledge structure and global research trends in gout: A bibliometric analysis from 2001 to 2021. Front. Public Health 10, 924676. 10.3389/fpubh.2022.924676 35844867 PMC9277182

[B38] Wu H. Cheng K. Guo Q. Yang W. Tong L. Wang Y. (2021). Mapping knowledge structure and themes trends of osteoporosis in rheumatoid arthritis: A bibliometric analysis. Front. Med. (Lausanne) 8, 787228. 10.3389/fmed.2021.787228 34888333 PMC8650090

[B39] Xie H. Cui Z. Wang L. Xia Z. Hu Y. Xian L. (2014). PDGF-BB secreted by preosteoclasts induces angiogenesis during coupling with osteogenesis. Nat. Med. 20, 1270–1278. 10.1038/nm.3668 25282358 PMC4224644

[B40] Yang K. Pei L. Wen K. Zhou S. Tao L. (2021). Investigating research hotspots and publication trends of spinal stenosis: A bibliometric analysis during 2000-2018. Front. Med. (Lausanne) 8, 556022. 10.3389/fmed.2021.556022 34354999 PMC8330839

[B41] Yang Z. Lin J. Li H. He Z. Wang K. Lei L. (2022). Bibliometric and visualization analysis of macrophages associated with osteoarthritis from 1991 to 2021. Front. Immunol. 13, 1013498. 10.3389/fimmu.2022.1013498 36268031 PMC9577295

[B42] You Y. Wang D. Liu J. Chen Y. Ma X. Li W. (2022). Physical exercise in the context of air pollution: An emerging research topic. Front. Physiol. 13, 784705. 10.3389/fphys.2022.784705 35295574 PMC8918627

[B43] Zhang J. Lin M. Huang Y. Wang Y. Huang T. Wu Z. (2022a). Harnessing hyaluronic acid for the treatment of osteoarthritis: A bibliometric analysis. Front. Bioeng. Biotechnol. 10, 961459. 10.3389/fbioe.2022.961459 36185454 PMC9516768

[B44] Zhang X. Cai G. Jones G. Laslett L. L. (2022b). Intravenous bisphosphonates do not improve knee pain or bone marrow lesions in people with knee osteoarthritis: A meta-analysis. Rheumatol. Oxf. Engl. 61, 2235–2242. 10.1093/rheumatology/keab786 34687305

[B45] Zhang X. Lai H. Zhang F. Wang Y. Zhang L. Yang N. (2021a). Visualization and analysis in the field of pan-cancer studies and its application in breast cancer treatment. Front. Med. (Lausanne) 8, 635035. 10.3389/fmed.2021.635035 33681260 PMC7926202

[B46] Zhang Y. Wang Y. Chen J. Cheng Q. Zhang B. Hao L. (2021b). The top 100 cited articles in osteonecrosis of the femoral head: A bibliometric analysis. Biomed. Res. Int. 2021, 1433684. 10.1155/2021/1433684 34462719 PMC8403054

[B47] Zhen G. Wen C. Jia X. Li Y. Crane J. L. Mears S. C. (2013). Inhibition of TGF-β signaling in mesenchymal stem cells of subchondral bone attenuates osteoarthritis. Nat. Med. 19, 704–712. 10.1038/nm.3143 23685840 PMC3676689

[B48] Zhu S. Zhu J. Zhen G. Hu Y. An S. Li Y. (2019). Subchondral bone osteoclasts induce sensory innervation and osteoarthritis pain. J. Clin. investigation 129, 1076–1093. 10.1172/JCI121561 PMC6391093 30530994

[B49] Zhu X. Chan Y. T. Yung P. S. H. Tuan R. S. Jiang Y. (2021). Subchondral bone remodeling: A therapeutic target for osteoarthritis. Front. Cell. Dev. Biol. 8, 607764. 10.3389/fcell.2020.607764 33553146 PMC7859330

